# Evaluation of competency and job satisfaction by positive human psychology among physical education teachers at the university level: A systematic review

**DOI:** 10.3389/fpsyg.2022.1084961

**Published:** 2022-12-20

**Authors:** Tingting Yan, Eng Wah Teo, Boon Hooi Lim, Bo Lin

**Affiliations:** ^1^Faculty of Sport and Exercise Sciences, Universiti Malaya, Kuala Lumpur, Malaysia; ^2^School of Physical Education, Henan Institute of Science and Technology, Xinxiang, China

**Keywords:** psychological health, physical exercise, physical education teachers, competency, job satisfaction

## Abstract

The study aimed to determine how a teacher’s competency at the university level related to professional commitment and job satisfaction. This systematic review intends to determine physical education teachers’ competency and job satisfaction. One of the main objectives of physical education programs is to increase various activities of the student, especially physical activities and participation in it. Students who participate in physical activities feel a variety of emotions, and these may enhance their mental and physical well-being. The study concerns the teachers’ satisfaction in effectively influencing and teaching the students. Job satisfaction refers to teachers’ feelings and positive attitudes toward their work. The five components of job satisfaction are pay, promotion, supervision, co-workers, and work itself. Job satisfaction is broken down into these five components. Work as a teacher is one of the most important components of a teacher’s or teacher’s success at work and one of the key criteria for the school’s achievement. Electronic libraries were explored, including Google Scholar, PubMed, Embase, Bing Academic, and Cochrane. Appropriate keywords were used for searching the literature. By applying certain inclusion and exclusion criteria, the final studies were selected for evaluation. The study’s findings showed that physical education instructors had high levels of teaching competence and moderate levels of job satisfaction. The findings demonstrated that job satisfaction and competency rise when degree levels rise, and relatedness, skill, and autonomy self-motivation ensue. Students’ motivation for physical education was greatly influenced by the teachers’ support, motivation, and mastery climate. The self-determination theory positively enhances physical education from the teacher’s perspective on PE, increasing instruction effectiveness.

## Introduction

Physical activity is the foundation and the primary teaching instrument in physical education. One of the main objectives of physical education programs is to increase pupils’ physical activity. Students who participate in physical activities feel a variety of emotions, and these may enhance their mental and physical well-being (Sparks et al., 2017; Oh and Song, 2021). Formal education and training can include physical education as a crucial component. Students also find physical education classes to be entertaining and satisfying. The educational system needs to be altered sometimes to strengthen its internal skills by selecting better inputs and a better structure with more suitable experiences. The outcomes of this system will meet the standards set by society for the educational system. Governments have used various tactics to achieve this goal, functioning to prepare teachers based on the fundamental competencies required for the profession of education should be considered (AlTobi et al., 2019). Modern education has developed to ensure that the educational system is responsive and adaptable to abrupt changes and the retraining of teachers and students at all stages and levels of education (Raj and Verma, 2018). Therefore, the education ministry has taken good care that can provide teachers with qualified scientific and educational backgrounds to understand the changes in society and adapt to their new roles, and this necessitates the development of teaching skills and competencies to allow the teacher to apply their area of expertise and instructional strategies (AlTobi et al., 2019).

The idea of competence is often used to imply quality or conforming to a standard. The idea of teacher competency encompasses the attitudes, information, and abilities that instructors ought to possess. Competencies results in better performance of the teachers in their respective jobs (Demir, 2015). A competent teacher is expected to perform their job efficiently in any particular situation teacher competence refers to a teacher’s ability to instruct effectively (De-Ketele, 1996; Bibi, 2005). According to Deakin and Turner (2008), a teacher’s competence is a composite collection of information, diverse abilities, understanding, beliefs, and attitudes that drive successful action to address the problem. According to Rychen and Salganik (2003), competence is the capacity to carry out complex acts with ease, accuracy, and adaptability. In the context of education, teacher competency is extremely important. It comprises three pillars (AlTobi et al., 2019).

**FIGURE 1:**
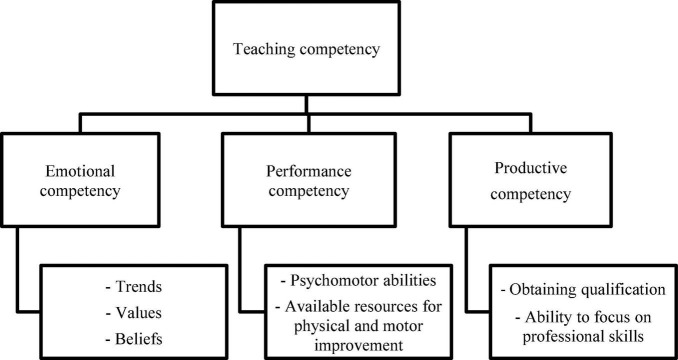
Teaching competency is made up of emotional competency, performing competencies, and productive competencies.

The following are the main components of teaching competencies (Figure 1):

1. Emotional competencies: These include an individual’s performance as well as their emotional behaviour, trends, values, and beliefs. These competencies encompass various topics, including an individual’s sensitivity, self-acceptance, and career direction.
2. Performing competencies: The abilities demonstrated by a person, which comprise psycho-motor abilities and resources for physical and motor development.
3. Productive competencies: A teacher’s productivity depends on the teacher’s skill training and qualifications to perform their task efficiently (AlTobi et al., 2019).

Educational competencies consist of three main categories: competency related to teaching in the classroom, competency related to undertaking or taking part in professional activities in school, and competency in moral contributing to society (Bhargava and Paty, 2010). Askar et al. (2008) outlined the educational competencies required of school instructors, which were divided into five categories, as shown in Table 1 below.

**TABLE 1:** The five categories of educational competencies.

Categories	Explanation
General competencies	The capacity for social and psychological integration and the capacity for personal, professional, and economic growth.
Specific competencies	This demonstrates adequate familiarity with and competence in the subject matter or body of knowledge to be taught.
Teaching-related capabilities	Identifying student differences, being aware of the standards for each educational level, and having the know-how and abilities to address students’ educational issues.
Cultural and social competencies	Understanding the social and cultural norms of the neighbourhood, as well as the function of educators in fostering social development.
Self-development competencies	The knowledge and ability to utilise the information sources required for career advancement through self-learning (Altaweel and AlJa’afreh, 2017).

The third study’s variable, job satisfaction, is an emotional situation related to how well or poorly one feels about one’s professional experiences (Dunn and Harris, 1998). For example, teachers’ dissatisfaction with the working environment and salary scale results in absenteeism and resignations. The five components of job satisfaction are pay, promotion, supervision, co-workers, and work itself. Job satisfaction is broken down into these five components. The extent of a person’s good thoughts towards their wage is referred to as their level of pay satisfaction. Their pay influences individuals’ job satisfaction, which improves employee performance (Skaalvik and Skaalvik, 2011). Poor employee satisfaction with an inefficient administrative process would result in reduced concentration at work, which may lead to poor performance of the employee (Cheema et al., 2015). Positive feedback, good two-way communication, and a preference for quality over quantity are all signs of satisfaction. Employee inspiration, motivation, and instructional effectiveness are all improved by effective supervision (Bakker and Bal, 2010). Co-workers greatly influence employee mood and behaviour. The level of job satisfaction is increased through improved communication between co-workers and bosses (Pearce, 1993). The importance of work in boosting job satisfaction cannot be overstated. Work satisfaction rises by supporting employees and encouraging learner growth (Luthans and Church, 2002).

Job satisfaction, which refers to a teacher’s feelings and positive attitudes towards their work as teachers, is one of the most important components of a teacher’s or teacher’s success at work, as well as one of the key criteria of the school’s achievement. Employees in any organisation perform better when they are satisfied with their work, especially when the source of this emotion is work rather than monetary rewards from the job (AlTobi et al., 2019). More skills than any other teaching position are needed to be a physical education teacher. His duties are varied, and the community looks up to him as a leader who can foster and preserve the general health of the next generation in the era of machines. There is some debate as to whether teaching physical education has become a more difficult job. One of the most important, contentious, and challenging subjects in psychology and behavioural management is job satisfaction (Binu et al., 2020). This research eventually demonstrated that the productivity of the teachers increases for a shorter time due to unique changes in the working environment (Skaalvik and Skaalvik, 2011).

**FIGURE 2:**
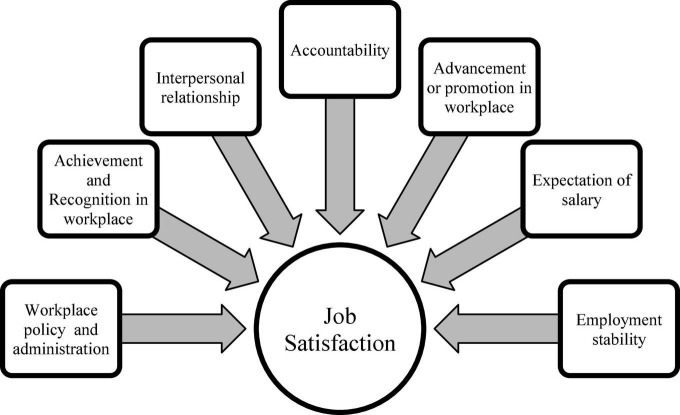
Elements that play a role in job satisfaction.

Several factors contribute to job satisfaction, which is traditionally seen as a continuum. Satisfaction among the teachers with their job depends on these elements (Figure 2). The absence of hygiene issues, like the absence of a cleaning service from time-to-time, did not affect job discontent or motivation, nor did it increase the presence of motivators. It was found that 14 factors either increased or decreased job satisfaction. Some of the notable factors are achievement, recognition, interpersonal relationships, accountability, advancement, pay, job stability, one’s personal life and status, one’s workplace environment, policy and administration, supervision, and the work itself are among the elements noted (Oh and Song, 2021).

## Methodology

**FIGURE 3:**
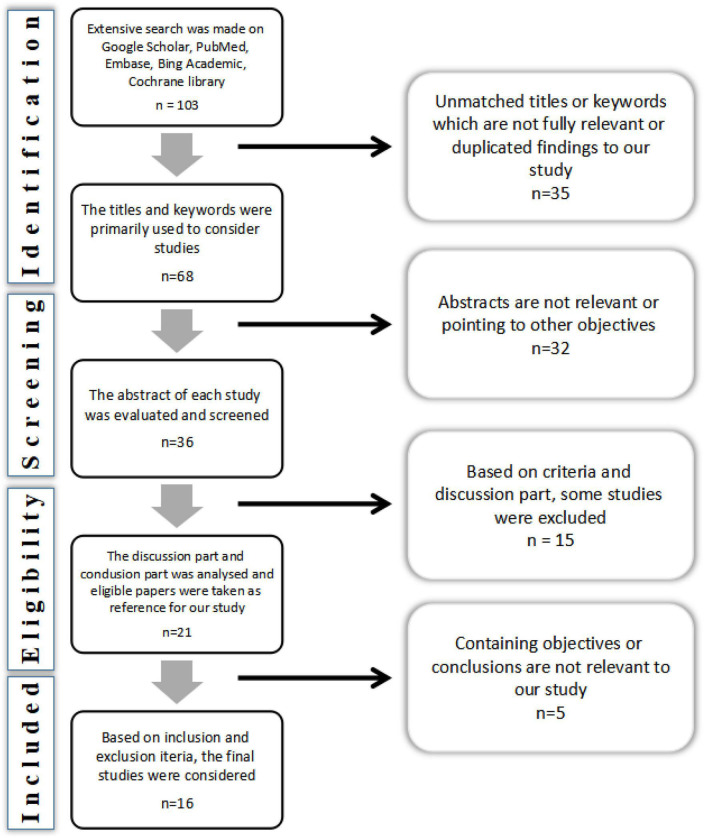
Preferred reporting items for systematic reviews and meta-analyses (PRISMA) diagram of the study.

Initially, the search was conducted through several electronic libraries. However, for this study, Google Scholar, PubMed, Embase, Bing Academic, and Cochrane libraries, were used to search for relevant literature. “Competency”, “job satisfaction”, and “physical education teachers” keywords were mainly used for searching. Specifically, the keyword formula used was (competency or performance or ability), (happiness or satisfaction or job satisfaction), (college level or college or university or educational institutes), and (sports teachers or physical education teachers). The inclusion and exclusion criteria were applied strictly. The included studies were also conducted between 2010 and 2022, which evaluated the impact of several factors on job satisfaction. Those studies that did not present a clear conclusion or did not discuss the effects comprehensively on job satisfaction were excluded. Good study selection requires a rigorous process; abstract screening is the first stage in that process. Figure 3 shows the PRISMA flowchart with details of the inclusion and exclusion of studies in this review.

## Results

According to the study’s findings, teacher-related sources of job satisfaction, like the gender of the teachers (Taherkhani, 2015), and perceived competence (Ntoumanis, 2001), seem to have a greater influence on teaching effectiveness (Table 2). There is a positive correlation between the degree held and the satisfaction levels. The higher the degree held at hand, the greater the level of work satisfaction. Teaching students the outdoor education (OE) curriculum enhances their skills while allowing teachers to participate in Physical Education Teacher Education (PETE) activities to enhance their sense of efficacy. Students’ motivation for physical education was greatly influenced by the teachers’ support, motivation, and efficiency. The self-determination theory enhances physical education positively from the teacher’s perspective on PE, increasing the effectiveness of instruction. The effectiveness of the instructor in delivering physical education was influenced by the factors like time, pressure, the choice given to the teachers, and the likely behaviour towards the teacher by the students.

**TABLE 2:** Summary of findings and conclusion of included studies in this review.

References	Findings	Conclusion
Ololube (2006)	A response rate of 69.1% was achieved, with a total of 690 of the 1,000 questionnaires distributed to the randomly chosen sample being returned. Six hundred and eighty of these 690 questions were considered suitable for analysis.	Since teachers are also dissatisfied with education policy and administrative structure, pay and material rewards, fringe benefits and advancement, and advancement opportunities, the survey results showed that teacher-related sources of job satisfaction appear to have a higher impact on teaching performance.
Taherkhani (2015)	The findings indicated that physical educators’ mean job happiness score is 156/26, with SD 17/68, representing their ideal level of job satisfaction. Male physical educators’ mean work satisfaction score was 129.54 ± 16.25 whereas female physical educators’ mean job satisfaction was 158.44 ± 19.55.	The study states that the job satisfaction level is higher in females, as stated in the findings. The levels of satisfaction are related to the degree level; the higher the degree, the higher level of job satisfaction.
Taylor et al. (2008)	The outdoor education (OE) curriculum was included in Physical Education Teacher Education (PETE) programs in a variety of residential settings, and they were assessed using the “Survey of Self-efficacy for Teaching Outdoor Education.”	Carrying out PETE programs with the OE curriculum improved their skills. So, to improve the teachers’ self-efficacy in teaching the OE, they are allowed to participate in the PETE programs, enhancing their ability to teach the OE.
Cox and Williams (2008)	This study’s goal was to examine how perceived competence, autonomy, and relatedness function as mediators in the relationship between social context elements and motivation in students studying physical education (*n* = 508).	The study concluded that the teacher support, motivation, and mastery climate were much more important and played an important role in motivating the students in physical education.
Rutten et al. (2012)	From 2,418 students in the sixth grade, data were gathered. In conducting analyses, bootstrapping was used.	The results showed that autonomy, competency, and relatedness, both by the physical education teacher and the physical school environment, promote autonomous motivation by satisfying the autonomous need.
Van Der Westhuizen et al. (2020)	The study’s findings are that, even in the face of difficulties like large classes, limited time, and a lack of equipment, the participants felt that their knowledge and understanding of physical education improved.	The study concluded that the in-service training based on the self-determination theory provides positive enhancement on physical education from the perception of the teacher on PE which shows a positive influence and increases the teaching effectiveness.
Cid et al. (2019)	This study has found that learning climate has the greatest influence on the positive motivation of teachers. However, autonomous motivation and intentions have roles to play. The study mainly highlighted that a positive teaching environment positively impacts psychological needs, ultimately improving students’ satisfaction.	The study concluded that a positive environment leads to higher job satisfaction due to fulfilling the teaching objectives. The working environment is the most important determinant of the teacher’s job satisfaction.
Pan (2014)	The research tools created by the researchers based on theories and existing instruments included the Teachers’ Self-Efficacy Scale, Learning Atmosphere Scale, Learning Motivation Scale, and Learning Satisfaction Scale for students.	The study concluded that the teachers’ self-efficacy impacted the students’ motivation, satisfaction, and atmosphere.
Zhang et al. (2011)	Two hundred eighty-five middle school students from the Southeast United States participated. They answered questions on their perceived need for teacher support, requirement satisfaction, and intrinsic motivation. A good match between the proposed model and the data was seen (RMSEA = 0.09; CFI = 0.96; NFI = 0.95; GFI = 0.97).	The study concluded that the intrinsic motivation in students is predicted by relatedness, perceived autonomy, and competence, which support the self-determination theory.
Oh and Song (2021)	The study found that the teacher’s support correlates with the psychological need of the student. The study has demonstrated that the higher relatedness support of the teachers would result in higher satisfaction of the student’s psychological needs, ultimately leading to positive motivation among the students. In physical education classes, the higher the teacher’s relatedness support, the higher the satisfaction of students’ psychological needs, which positively impacts their motivation. The teachers’ job satisfaction depends on fulfilling the jobs’ objectives, which are attributed to the teaching achievements. This achievement also depends on the teachers’ and students’ communication and relationship.	The study has concluded that both relatedness and autonomy support of the teachers are important. The teachers are more satisfied if their tasks can be fulfilled successfully.
Binu et al. (2020)	There is no significant difference in job satisfaction based on gender. However, job satisfaction was found to be highest among the government school teachers, while the schools under the affiliation of the central board, had the lowest job satisfaction.	The study concluded that job satisfaction largely depends upon the salary scale and working conditions, not gender.
Hölbl et al. (2018)	It provides a proper statement of the treatment populations that are essential, the intervention strategies that are of significance, and the outcomes.	The data is used to choose research to include in the analysis.
Hanelt et al. (2020)	A systematic review can be done based on a research approach already set up. People need to discuss things like the quality and range of the research used and the potential impacts of judgement.	This way, the level of quality of the exploration can be evaluated. Research approaches might also look at the Cochrane Controlled Trials Register (CCTR), many digital resources, and able to register experiments that are not in the CCTR.
Winkelhaus and Grosse (2020)	After a comprehensive survey, all potential research methods that have been found ought to be evaluated and see whether they are decent enough to be included.	It must be discussed how to apply specific criteria to categories of workers, treatments, results, research settings, and the performance of the methods.
Agbo et al. (2019)	The outcomes of previous studies that can be connected are then provided together to consider an “overall assessment” of how well the action applies in the real world.	The result can always be done qualitatively or stronger by using facts and figures to combine the information from numerous analyses into a single measurement of all of it.
Ives and Castillo-Montoya (2020)	Meta-analysis has been the technique of putting together a lot of scientific techniques. In a meta-analysis, the performance of different research is kept together to use the “inverse variance method.”	The outcomes of the meta-analysis and statistical sharing of the research findings also must be defined, discussed, and the implications of the findings or more studies must be pointed out.

## Discussion

Substantially, job satisfaction and encouragement, professional experience skills, centre competencies, educational resources, and instructional strategies are the factors that genuinely determine educational performance and achievement. This is important for the continued growth of academic systems around the world. Since teachers are also unsatisfied with educational practices and administration, pay and benefits packages, material rewards and advancement, and advancement opportunities, the survey results showed that teacher-related sources of job satisfaction appear to have a significantly larger impact on teaching performance (Zhang et al., 2011).

A study was conducted whose findings indicated a significant positive relationship between physical education instructors’ inventiveness and their contentment with their jobs. While there was no significant difference in teachers’ levels of creativity, there was a significant variation in their levels of job satisfaction. Instructors with higher degrees are more satisfied with their jobs and are more creative (Taherkhani, 2015).

By employing motivating techniques, physical education instructors can affect their pupils’ ability to make decisions for themselves. The relationships between the perception of job pressure, perceptions of student autonomy, teachers’ autonomous awareness, psychological requirement satisfaction, and self-determination to teach, and teachers disclosed the use of three motivational techniques (providing a purposeful rationale, supplying instrumental assistance and support, and obtaining an understanding of the students). According to a model backed by structural equation modelling, teacher autonomy orientation, perceptions of student autonomy, and felt job pressure all anticipated teacher psychological demand satisfaction, which in turn had a favourable impact on teacher self-determination (Derlin and Schneider, 1994; Chaplain, 1995). The final correctly forecasts the application of all three tactics. Teachers’ psychological needs positively impacted their methods for comprehending pupils and providing them with support and assistance. Elements that affect teacher motivation may also indirectly impact how they motivate pupils (Taylor et al., 2008).

The good effects of perceived competence, independence, and mastery climate and the unfavourable impact of performance environment on students’ motivation in physical training are demonstrated by research. Less study has been done on how connections in this environment-specifically, perceived support from teachers and relatedness-affect student motivation. According to the findings of structural equation modelling, mastery climate was directly correlated with self-determined motivation, and perceived competence, independence, and relatedness partially mediated the relationship between self-determined motivation and perceived teacher support (Cox and Williams, 2008). Another study demonstrated that views of autonomy and competence mediated the relationship between autonomous motivation and the need for the PE teacher’s support. The impression of autonomy also mediated the relationship between the actual education system and autonomous motivation. These results imply that in addition to the physical education instructor, the physical school climate can also encourage autonomous motivation by meeting the desire for autonomy (Rutten et al., 2012).

Physical Education (PE) has many implementation difficulties in South Africa even though its health advantages are widely acknowledged, one of which is the dearth of certified PE teachers. Suppose the fundamental psychological demands of autonomy, competence, and relatedness are met. In that case, in-service training can improve PE teachers’ motivation and views of the subject, according to the Self-determination Theory (SDT). The impact of an in-service support and training program based on the SDT principles on teachers’ attitudes towards physical education and the effectiveness of their instruction. The results showed that the respondents feel that their understanding and knowledge of physical education have improved even in the face of difficulties like large classes, little time, and a lack of equipment. They also felt they were more effective in applying various organisational, assessment, and teaching strategies in the PE class. Positive influences were cited from the suggested activity topics and the encouragement from teachers and fellow students. It was determined that PE in-service training and support programs that meet the participants’ basic psychological needs within the SDT framework could positively affect teachers’ perceptions of PE and their efficacy as teachers, which will help to improve PE in South Africa (Van Der Westhuizen et al., 2020).

Physical education (PE) is widely considered to have the ability to significantly improve public health by fostering a good attitude towards physical activity and by encouraging fitness programs that are related to health. If children are not encouraged to participate fully in their PE courses, these initiatives will be restricted in their effectiveness. Perceived competence was the main psychological mediator, according to an SEM study. Positive outcomes were associated with intrinsic motivation, whereas bad outcomes were associated with external regulation and motivation. The results highlight the significance of personal motivation and perceived competence in required physical activity (Ntoumanis, 2001).

Pan stated in his study on senior high school physical education which was aimed at confirming the links between teachers’ self-efficacy, students’ learning motivation, learning environment, and learning satisfaction, found that learning motivation, learning environment, and learning satisfaction were all influenced by the self-efficacy of physical education teachers. Teachers’ self-efficacy also influences learning satisfaction indirectly and favourably through learning motivation and environment (Pan, 2014). A study has been conducted to test a structural model of predicted relationships between perceived required assistance from instructors (independence support, competence assistance, and relatedness assistance), psychological requirement satisfaction (independence, relatedness, and competence), internal motivation, and physical activity using the self-determination theory as a framework. The link between the need for assistance and physical activity was mediated by need satisfaction and personal drive. The notions of autonomous motivation, competence, and relatedness are the elements that support students’ intrinsic motivation and eventually positively predict their physical activity. The results confirmed the self-determination theory’s theoretical precepts (Zhang et al., 2011).

Another study was conducted in which a questionnaire was created to measure the self-efficacy of physical education teachers to teach classes where their pupils participated in high levels of physical exercise. When teachers did not have sufficient time to teach, the time factor was a good indicator of their effectiveness. When teaching was challenging due to a shortage of space, the space component reflected teachers’ judgements of their efficacy. The institution factor, which indicated teachers’ confidence in their ability to overcome a lack of institutional support, was also made up of questions. The effectiveness of teachers in handling pupils who did not value or enjoy physical activity was reflected in the student factors (Martin and Kulinna, 2003).

In a study titled examining factors that influence instructors’ intentions to lead physically active physical education lessons, spending at least 50% of the class time having students engage in moderate to strenuous physical activity was the goal. A model was investigated with the hypothesis that teachers’ intentions were influenced by subjective norms, attitudes, perceived behavioural control, and self-efficacy (Mahmud et al., 2012). This study was based on reasoned action, planned behaviour, and self-efficacy. The theories of planned behaviour and planned conduct were supported by hierarchical regression analyses, which accounted for 66% of the variance in intention caused by attitude and subjective norm as well as their interaction effects. In the study, Self-efficacy theory and the influence of perceived behavioural control were not supported (Martin et al., 2001). Physical Education Teacher Education (PETE) programs must target OE outcomes because self-efficacy has been identified as impacting teacher effectiveness and the rising popularity of outdoor education (OE) content taught in physical education contexts. This study found a significant improvement in self-efficacy scores across all content areas from the pre-test to the post-test (OE skills, dynamic group skills, and models and theories) and also found that in general, OE programs significantly changed the self-efficacy scores. PETE students’ self-efficacy for teaching OE increased due to their participation in the program, which might enhance their capacity to do so in physical education settings in the future (Hovey et al., 2019).

## Conclusion

The study concludes that teaching competencies be made available to instructors, included in programs for preparing and qualifying teachers, and trained to address the moral, financial, and social needs of physical education teachers. The study has effectively achieved its objectives to determine a teacher’s competency at the university level related to professional commitment and job satisfaction. The outcome also recommends undertaking additional research to examine instructors’ teaching abilities and job satisfaction at various educational levels and in various fields. However, further subcomponents were not detected. The gender of the research group appeared to be a subcomponent in motivating interaction. Female students were shown to contribute more and be more willing to participate in physical education sessions than the opposite gender. The level of competence and job satisfaction also increases with a higher degree. The teachers’ self-efficacy increases by conducting physical education teacher education programs which increase the teacher’s interest in teaching outdoor education to the students. The role of the teacher in motivating and supporting the students will increase the students’ interest in participating in physical education (Alvarenga et al., 2017). This study has some limitations, such as the availability of studies in limited countries, so this study could not consider studies from numerous countries for effective discussion. However, this study highlighted important points that will effectively improve teaching programs for physical education teachers at the university level.

## Data availability statement

The original contributions presented in this study are included in this article/supplementary material, further inquiries can be directed to the corresponding authors.

## Author contributions

BHL was responsible for helping revise the manuscript, providing edits for the revision, and proofreading. TY was responsible for manuscript writing. EWT and BL were responsible for manuscript validation. All authors contributed to the article and approved the submitted version.

## References

[B1] Agbo C. C. Mahmoud Q. Eklund J. (2019). Blockchain technology in healthcare: A systematic review. *Healthcare* 7:56. 10.3390/healthcare7020056 30987333 PMC6627742

[B2] Altaweel A. AlJa’afreh A. (2017). Competencies in physical education teaching: An investigation of teachers’ perceptions in the Southern Governorates of Jordan. *J. Stud. Educ.* 7 54–68.

[B3] AlTobi A. S. Al-Shboul M. Aldoulat A. Al-Halalsheh N. Aldoulat H. (2019). Teaching competencies and job satisfaction among basic education teachers. *Modern Appl. Sci.* 13 25–36.

[B4] Alvarenga C. E. A. Ginestié J. Brandt-Pomares P. (2017). How and why Brazilian and French teachers use learning objects. *Educ. Inform. Technol.* 22 1973–2000.

[B5] Askar P. Altun A. Ilgaz H. (2008). “Learner satisfaction on blended learning,” in *Proceedings of the E-leader conference* (Krakow).

[B6] Bakker A. B. Bal M. P. (2010). Weekly work engagement and performance: A study among starting teachers. *J. Occupat. Organ. Psychol.* 83 19–34.

[B7] Bhargava A. Paty M. (2010). Quintessential competencies of a teacher: A research review. *Int. J. New Trends Educ. Their Implicat.* 1 7–18.

[B8] Bibi S. (2005). *Measuring poverty in a multidimensional perspective: A review of literature. PEP working paper No. 2005-07.* 1–38.

[B9] Binu G. V. John J., Thomas M. (2020). A study on job satisfaction among physical education teachers working in government, aided and CBSE schools in Kerala. *Int. J. Creative Res. Thoughts* 8 41–62.

[B10] Chaplain R. P. (1995). Stress and job satisfaction: A study of english primary school teachers. *Educ. Psychol.* 15 65–81.

[B11] Cheema S. Akram A. Javed F. (2015). Employee engagement and visionary leadership: Impact on customer and employee satisfaction. *J. Bus. Stud. Q.* 7:139.

[B12] Cid L. Pires A. Borrego C. Duarte-Mendes P. Teixeira D. S. Moutão J. M. (2019). Motivational determinants of physical education grades and the intention to practice sport in the future. *PLoS One* 14:e0217218. 10.1371/journal.pone.0217218 31120973 PMC6592572

[B13] Cox A. Williams L. (2008). The roles of perceived teacher support, motivational climate, and psychological need satisfaction in students’ physical education motivation. *J. Sport Exer. Psychol.* 30 80–102. 10.1123/jsep.30.2.222 18490792

[B14] Deakin N. O. Turner C. E. (2008). Paxillin comes of age. *J. Cell Sci.* 121, 2435–2444. 10.1242/jcs.018044 18650496 PMC2522309

[B15] De-Ketele J. M. (1996). Evaluation of educational achievement: What? Why? For what. *J. Tunis. Sci. Educ.* 23, 17–36.

[B16] Demir E. (2015). Evaluation of professional personality competence of physical education teachers working in secondary schools by students. *J. Educ. Train. Stud.* 4 133–149.

[B17] Derlin R. Schneider G. T. (1994). Understanding job satisfaction: Principals and teachers, urban and suburban. *Urban Educ.* 29 63–88, 10–21.

[B18] Dunn R. J. Harris L. G. (1998). Organisational dimensions of climate and the impact on school achievement. *J. Instruct. Psychol.* 25 115–134. 10.1007/s10464-015-9733-z 26099299

[B19] Hanelt A. Bohnsack R. Marz D. Antunes Marante C. (2020). A systematic review of the literature on digital transformation: Insights and implications for strategy and organizational change. *J. Manag. Stud.* 58 1159–1197. 10.1111/joms.12639

[B20] Hölbl M. Kompara M. Kamisalic A. Nemec Zlatolas L. (2018). A systematic review of the use of blockchain in healthcare. *Symmetry* 10:470. 10.3390/sym10100470

[B21] Hovey K. Niland D. Foley J. T. (2019). The impact of participation in an outdoor education program on physical education teacher education student self-efficacy to teach outdoor education. *J. Teach. Phys. Educ.* 39 120–135.

[B22] Ives J. Castillo-Montoya M. (2020). First-generation college students as academic learners: A systematic review. *Rev. Educ. Res.* 90:003465431989970. 10.3102/0034654319899707

[B23] Luthans F. Church A. H. (2002). Positive organisational behavior: Developing and managing psychological strengths [and executive commentary]. *Acad. Manag. Execut. (1993-2005)* 16 13–29.

[B24] Mahmud R. Ismail M. A. H. Rahman F. A. Kamarudin N. Ruslan A. R. (2012). Teachers’ readiness in utilising educational portal resources in teaching and learning. *Proced. Soc. Behav. Sci.* 64 484–491.

[B25] Martin J. J. Kulinna P. H. (2003). The development of a physical education teachers’ physical activity self-efficacy instrument. *J. Teach. Phys. Educ.* 22 219–232.

[B26] Martin J. J. Kulinna P. H. Eklund R. C. Reed B. (2001). Determinants of teachers’ intentions to teach physically active physical education classes. *J. Teach. Phys. Educ.* 20 30–48.

[B27] Ntoumanis N. (2001). A self-determination approach to the understanding of motivation in physical education. *Br. J. Educ. Psychol.* 71 66–81. 10.1348/000709901158497 11449934

[B28] Oh J. Song J. H. (2021). The relationship between physical education teachers’ relatedness support and students’ psychological needs and study motivation. *Turkish J. Comput. Math. Educ.* 12 1056–1064. 10.1080/17461391.2016.1276968 28100122

[B29] Ololube N. P. (2006). Teachers job satisfaction and motivation for school effectiveness: An assessment. *Essays Educ.* 18:9.

[B30] Pan Y. H. (2014). Relationships among teachers’ self-efficacy and students’ motivation, atmosphere, and satisfaction in physical education. *J. Teach. Phys. Educ.* 33 41–60. 10.1186/s12913-016-1423-5 27409075 PMC4943498

[B31] Pearce J. L. (1993). Toward an organisational behavior of contract laborers: Their psychological involvement and effects on employee co-workers. *Acad. Manag. J.* 36 1082–1096.

[B32] Raj M. Verma J. (2018). Teaching competence and organisational commitment among high school teachers. *Int. J. Res. Anal. Rev.* 5 101–119.

[B33] Rutten C. Boen F. Seghers J. (2012). How school social and physical environments relate to autonomous motivation in physical education: The mediating role of need satisfaction. *J. Teach. Phys. Educ.* 31 101–119.

[B34] Rychen D. S. Salganik L. H. (eds) (2003). *Key competencies for a successful life and well-functioning society.* Vol. 12. Göttingen: Hogrefe Publishing, 91–109.

[B35] Skaalvik E. M. Skaalvik S. (2011). Teacher job satisfaction and motivation to leave the teaching profession: Relations with school context, feeling of belonging, and emotional exhaustion. *Teach. Teach. Educ.* 27 88–92.

[B36] Sparks C. Lonsdale C. Dimmock J. Jackson B. (2017). An intervention to improve teachers’ interpersonally involving instructional practices in high school physical education: Implications for student relatedness support and in-class experiences. *J. Sport Exer. Psychol.* 39 233–241. 10.1123/jsep.2016-0198 28787252

[B37] Taherkhani E. (2015). Review of relation between creativity and job’s satisfaction of physical education teachers. *Cumhuriyet Üniversitesi Fen Edebiyat Fakültesi Fen Bilimleri Dergisi* 36 279–287.

[B38] Taylor I. M. Ntoumanis N. Standage M. (2008). A self-determination theory approach to understanding the antecedents of teachers’ motivational strategies in physical education. *J. Sport Exer. Psychol.* 30 102–118. 10.1123/jsep.30.1.75 18369244

[B39] Van Der Westhuizen S. J. Du Toit D. Van Der Merwe N. (2020). Effects of an in-service training and support programme on teachers’ perceptions of physical education and their teaching effectiveness. *Afr. J. Phys. Activity Health Sci. (AJPHES)* 26(Suppl. 1) 97–112.

[B40] Winkelhaus S. Grosse E. (2020). Logistics 4.0: A systematic review towards a new logistics system. *Int. J. Prod. Res.* 58 18–43. 10.1080/00207543.2019.1612964

[B41] Zhang T. Solmon M. A. Kosma M. Carson R. L. Gu X. (2011). Need support, need satisfaction, intrinsic motivation, and physical activity participation among middle school students. *J. Teach. Phys. Educ.* 30 77–89.

